# Metabolic Dysfunction and Alcohol-Associated Liver Disease: A Narrative Review

**DOI:** 10.14309/ctg.0000000000000828

**Published:** 2025-02-19

**Authors:** Nicholas Dunn, Naim Al-khouri, Ismail Abdellatif, Ashwani K. Singal

**Affiliations:** 1 Department of Medicine, Kansas University Medical Center, Kansas City, Kansas, USA;; 2 Department of Hepatology, Arizona Liver Health, Phoenix, Arizona, USA;; 3 Department of Medicine, Cleveland Clinic, Cleveland, Ohio, USA;; 4 Department of GI Hepatology and Nutrition, University of Louisville Health Sciences Center, Louisville, Kentucky, USA;; 5 Trager Transplant Center at Jewish Hospital, Louisville, Kentucky, USA;; 6 Robley Rex VA Medical Center, Louisville, Kentucky, USA.

**Keywords:** MetALD, MASLD, ALD, cirrhosis

## Abstract

The term steatotic liver disease (SLD) is now used to describe conditions involving fat accumulation in the liver. SLD term includes a spectrum of defined and less defined disorders: metabolic dysfunction-associated SLD (MASLD), alcohol-associated liver disease (ALD), and metabolic and ALD (Met-ALD), where both cardiometabolic risk factors, such as obesity, diabetes, or dyslipidemia, and alcohol consumption function in disease development and progression. Met-ALD is defined as liver disease in men with at least 1 cardiometabolic risk factor who also consume 210–420 g of alcohol per week (approximately 30–60 g per day), whereas for women, it is defined as at least 1 cardiometabolic risk factor in addition to consumption of 140–350 g of alcohol per week (approximately 20–50 g per day). This level of alcohol intake exceeds the thresholds traditionally used to exclude alcohol as a contributing factor in MASLD, but it remains below the levels typically associated with classic ALD. Met-ALD is estimated to affect about 17 million people in the United States It is a unique disease with the risk of cirrhosis, hepatocellular carcinoma, and mortality different from those with MASLD or ALD. Its treatment relies mainly on weight loss, alcohol abstinence, and control of cardiometabolic risk factors. Novel medications such as glucagon-like peptide-1 agonists and fibroblast growth factor s21 analogs may be promising future therapies for the treatment of Met-ALD.

## INTRODUCTION

**Figure 1.**
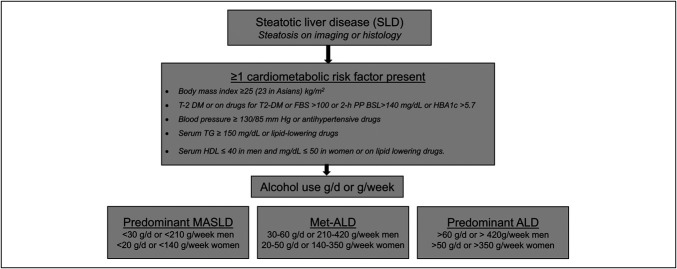
New nomenclature and definition of subtypes of SLD. ALD, alcohol-associated liver disease; HDL, high-density lipoprotein; MASLD, metabolic dysfunction-associated SLD; Met-ALD, metabolic dysfunction ALD; SLD, steatotic liver disease; TG, triglyceride.

With the perception of stigma associated with the words “Fatty” and “Alcoholic” in nonalcoholic fatty liver disease (1), the hepatology community using the multisociety Delphi consensus suggested to change the name of fatty liver disease to steatotic liver disease (SLD) and nonalcoholic fatty liver disease to metabolic dysfunction-associated SLD (MASLD) (2,3). According to this new nomenclature, SLD includes MASLD, alcohol-associated liver disease (ALD), and metabolic and ALD (Met-ALD). All these subtypes of SLD have a common feature of the presence of at least 1 cardiometabolic risk factor (CMRF) but differ based on the amount of alcohol use (Figure 1). Alcohol use <20 g/d in women and <30 g/d in men is defined as predominant MASLD, whereas alcohol use of 20–50 and 30–60 g/d in women and men, respectively, defines Met-ALD. As alcohol use is typically reported by patients as drinks, with varying definitions of 1 drink equivalence in gm of pure alcohol, this new nomenclature brings a strength of harmonizing the alcohol quantification worldwide, with use of the World Health Organization criterion of 10 g/d of pure alcohol equivalent to a drink.

Patients with SLD without CMRF can be stratified to other less important causes such as medication-induced SLD, other rare miscellaneous disorders, and cryptogenic SLD (3). Other than removing stigmatizing language, this change in nomenclature provided more accurate description of the etiology of the liver disease (3,4). Moreover, the introduction of Met-ALD is a giant leap in refining the understanding of liver diseases characterized by both metabolic dysfunction and alcohol consumption (4). The objective of this new classification was to provide diagnostic clarity on the subset of patients with metabolic risk factors and intermediate alcohol use, hence simplifying patient categorization and improving clinical management.

Met-ALD refers to liver disease where both CMRF (Figure 1) and alcohol consumption interact in disease development and progression (4). This shift in definition emphasizes the importance of recognizing the impacts of alcohol and metabolic dysfunction that contribute to liver injury. In addition, a more nuanced understanding of how these factors combine to accelerate liver disease progression is established. The inclusion of Met-ALD as a SLD opens a new door for patients who would otherwise be excluded from MASLD-predominant and ALD-predominant liver diseases (4,5).

Management of Met-ALD requires a comprehensive approach to address risk factors with lifestyle interventions targeting controlling alcohol intake and CMRF. Specific pharmacological interventions can supplement the management with glucagon-like receptor-1 (GLP-1) agonists (semaglutide, tirzepatide) and thyroid hormone receptor-beta agonist resmetirom [7]. Another potential target for medical therapy is fibroblast growth factor 21 (FGF21) with FGF21 analogs such as efruxifermin and pegozafermin showing promising results [8]. The aim of this article was to provide a comprehensive review on Met-ALD nomenclature, prevalence, healthcare burden, prognosis, up-to-date recommendations on its management, and recommendations for future directions in its research.

## CHALLENGES IN DIAGNOSIS OF Met-ALD

An introduction of a new entity of Met-ALD brings its own challenges and opportunities (6), for example, the diagnosis of relies on the presence of CMRF and the degree of alcohol intake. Based on that, for men, Met-ALD is defined as liver disease in individuals with at least 1 CMRF who also consume 210–420 g of alcohol per week (approximately 30–60 g per day), while for women, it is defined as at least 1 CMRF in addition to consumption of 140–350 g of alcohol per week (approximately 20–50 g per day) (2,3). This level of alcohol intake exceeds the thresholds traditionally used to exclude alcohol as a contributing factor in MASLD, but it remains below the levels typically associated with classic ALD. Within SLD, there is a continuum where the condition can be seen to be MASLD predominant or ALD predominant (Figure 1).

The diagnosis of MASLD, Met-ALD, and ALD subtypes of SLD requires a careful and accurate documentation of alcohol use by the individual. However, the self-reported consumption of alcohol may not be accurately reported because of recall bias (7). There is a clinical unmet need for alcohol use biomarkers such as phosphatidyl ethanol (PeTH) levels correlating with the amount of alcohol use if the individual is actively using alcohol. With a window of detection of PeTH over the preceding 6–8 weeks, it becomes challenging if the alcohol use is outside of this window, which calls for models using clinical and/or biomarker data to capture the Met-ALD as a subtype of SLD (8). Furthermore, even if the alcohol use information is accurately extracted, there is no *ICD-10* code for Met-ALD subtype of SLD, making it challenging to capture the true prevalence of the disease using electronic medical records databases.

### Assessment of fibrosis risk

The use of noninvasive tests for assessment of fibrosis stage is also challenging in patients with Met-ALD. There is standard cutoff used and recommended for patients with MASLD and ALD (9,10). However, it remains unclear whether to use MASLD or ALD cutoffs in patients with Met-ALD. In a recent study on 7,918 patients with SLD (5.9% Met-ALD), FIB-4 using the same cutoff as for MASLD had c-statistics of 0.85 in identifying advanced fibrosis defined with liver stiffness measurement of >3.6 kPa on MR elastography, with a sensitivity of 71.3%, specificity of 77.4%, negative predictive value of 99.4%, but a positive predictive value of only 4.6% (11). In one study, the authors showed that Steatosis-Associated Fibrosis Estimator (SAFE) score, a noninvasively assessed biomarker, accurately predicted mortality in the 3 SLD subtypes. The score, in its 3 tiers, namely SAFE <0 (low risk), 0–100 (intermediate risk), and >−100 (high risk), was able to stratify all 3 groups according to their probability of mortality. Compared with low-risk SAFE scores, intermediate-risk and high-risk SAFE scores were associated with a 31% and 90% increase in mortality, respectively. On the other hand, with the FIB-4 score, only the highest stratum (FIB-4 >−2.67) was significantly associated with an increased risk of mortality, with a 53% risk increase (12).

## EPIDEMIOLOGY AND HEALTHCARE BURDEN

The prevalence of CMRF and alcohol use, the 2 most common causes of SLD, is increasing in the general population, with a consequent increase in the prevalence of Met-ALD. Recent epidemiological studies have shed light on the prevalence in the United States, especially in primary settings (13,14).

Met-ALD is estimated to affect about 17 million people in the United States A recent study using the National Health and Nutrition Examination Survey (NHANES) database (2017–2020) showed that about 90% of patients with ALD have at least 1 CMRF (13). In this study, 9,698 adults completed a transient elastography examination. The age-adjusted prevalence of SLD among US adults was found to be 37.87% (95% CI, 35.13%–40.69%). MASLD prevalence was 32.45% (95% CI, 29.82%–35.18%) and Met-ALD prevalence was 2.56% (95% CI, 1.91%–3.41%), whereas ALD prevalence was 1.17% (95% CI, 0.71%–1.92%) (13). Using liver stiffness measurements (LSM), the authors determined clinically significant and advanced fibrosis with cutoff values of 8.6 and 13.1 kPa, respectively (13). Among patients diagnosed with MASLD, the age-adjusted prevalence of clinically significant fibrosis (LSM, >8.6 kPa) was 20.86% (95% CI, 17.12%–25.18%), and for advanced fibrosis (LSM, >13.1 kPa), it was 8.98% (95% CI, 6.43%–12.4%). By contrast, among the patients with Met-ALD, the age-adjusted prevalence for significant fibrosis (LSM, >8.6 kPa) stood at 13.27% (95% CI, 6.76%–24.39%), and for advanced fibrosis (LSM, >13.1 kPa), it was 5.47% (95% CI, 2.44%–11.78%). After multivariate logistic regression, the authors concluded that Met-ALD was not associated with a higher risk of significant or advanced fibrosis when compared with patients with MASLD (13).

Although Met-ALD is far less common than MASLD, it has been shown to have a higher risk of liver cirrhosis (hazard ratio [HR]: 1.72 vs 1.30, *P* < 0.0001) and hepatocellular carcinoma (HCC) (HR: 1.83 vs 1.31, *P* < 0.0001) compared with MASLD (15). On the other hand, a study by Sripongpun et al (12) demonstrated that ALD had a significantly lower overall survival compared with MASLD (*P* = 0.004), whereas there was no difference between Met-ALD and MASLD (*P* = 0.165). These figures also highlight the importance of distinguishing Met-ALD from MASLD, considering that moderate alcohol intake in people with metabolic dysfunction ushers in peculiar challenges relating to disease progression and management.

## CLINICAL FEATURES AND NATURAL HISTORY

Although both ALD and MASLD share many pathological features of steatosis and steatohepatitis, the clinical course and prognosis of these diseases differ significantly. It is known that ALD is more aggressive, with a worse prognosis compared with MASLD (16,17). In addition, ALD presents itself at later stages compared with MASLD (16). Patients with ALD are also at a greater risk of developing cirrhosis, HCC, and cirrhosis complications with portal hypertension such as ascites, variceal bleeding, and hepatic encephalopathy (16).

A retrospective comparative single-center study showed that patients with ALD more often present with cirrhosis or its complications including HCC compared with MASLD (46% vs 12% *P* value < 0.001) (16). On logistic regression analysis, diagnosis of ALD was associated with the presence of cirrhosis by over 3-fold (OR: 3.3 [1.3–8.2]). Patients with ALD also had a higher Model of End-Stage Liver Disease score (13 ± 7 vs 8 ± 8; *P* < 0.0001) and worse liver transplant (LT)-free survival (90 vs 95%; *P* = 0.038).

The clinical course and prognosis of ALD, compared with MASLD, underscore a careful difference in diagnosis and treatment. Though alcohol plays the major part in the rapid progress of ALD, the recent development of Met-ALD nomenclature has brought attention to the subset of liver diseases where both metabolic dysfunction and alcohol consumption contribute significantly to liver injury (4,6).

### Interaction of alcohol and cardiometabolic risk factors

A key metabolic contributor in Met-ALD is insulin resistance resulting in steatosis (18). Another interesting study, using the NHANES database (19), showed the effect of alcohol on CMRF especially hypertension and triglycerides. This raises the concern for overestimation of Met-ALD in the population. Hence, it is extremely important to document the quantity of alcohol use for better discrimination of 3 subtypes of MASLD (13).

Data are emerging on the damaging effects of alcohol on the progression of fibrosis even in patients with MASLD, a group with safer use of alcohol (<20 g/d in women and <30 g/d in men). In a population-based prospective study on 3,959 patients with MASLD, 14% reported moderate alcohol consumption (10–13 drinks per week in women and 10–20 per week in men); a dose-dependent increase in fibrosis risk and at-risk metabolic dysfunction-associated steatohepatitis (MASH) was observed with the number of drinks of alcohol consumed (20). The odds of at-risk MASH (FAST score >0.35) were 1.7 and 3.84 folds higher with low alcohol use (5–9 drinks/wk) and moderate (10–20 drinks/wk) as compared with very low level of alcohol use (0–4 drinks/wk). In MASLD, patients were shown to increase the risk of advanced disease to a level like that observed in Met-ALD (defined as 14–35 drinks/wk in women and 21–42 drinks/week in men). Similar odds for significant fibrosis (LSM >=8 kPa) were 1.53 and 2.71, respectively. Odds for at-risk MASH and for significant fibrosis in Met-ALD were 4.84 and 4.14, respectively, suggesting a dose-response negative impact of alcohol on liver health in patients with SLD. This suggests that there is no safe limit of alcohol use in MASLD subtype of SLD. Furthermore, the effective treatment of CMRF improves liver disease in all the 3 subtypes of SLD (21).

### Long-term risk of cirrhosis and complications

Despite the fact the Met-ALD carries a similar prevalence of liver fibrosis as MASLD, recent evidence suggests that it carries higher long-term risk of cirrhosis and HCC (15). In a prospective study that included 332,175 adults followed up for a median of 16 years by Chen et al, the incidence of cirrhosis in the Met-ALD group was 145.6 per 100,000 person-years, compared with 95.4 per 100,000 person-years in MASLD and 282 per 100,000 person-years in ALD (HR, 1.72 vs 1.30 vs 2.82, respectively, *P* value < 0.0001) (15). This further suggests that although Met-ALD does not progress as aggressively as ALD, the risk of cirrhosis is much higher when compared with MASLD, suggesting that even moderate consumption of alcohol contributes immensely to accelerating the progression in liver dysfunction cases because of metabolic dysfunction.

In the same study, the incidence population rate of HCC among Met-ALD was 52.7/100,000 person-years, which was significantly higher than that in MASLD (30.7/100,000 person-years), but lower than that in ALD (69.0/100,000 person-years), with respective adjusted HR of 1.31, 1.52, and 1.83 in MASLD, Met-ALD, and ALD, respectively, in a multivariable regression model analysis (*P* < 0.0001). These data suggest that more accustomed surveillance should be performed on patients with Met-ALD owing to higher metabolic and alcohol-derived risk factors relative to ALD and MASLD alone (15).

### Long-term risk of overall and liver-related mortality

In a study using NHANES database on 9,939 patients (Met-ALD in 2.3%, MASLD in 30%, and ALD in 1%) (12), compared with MASLD, those with ALD had lower overall survival (*P* = 0.004), but this was not observed in individuals with Met-ALD (*P* = 0.165). However, after adjusting for other variables in a multivariable model, compared with MASLD, the HR for mortality was 16% and 57% higher in Met-ALD and ALD, respectively. In the same study, compared with participants without SLD, MASLD, Met-ALD, and ALD were associated with 16%, 33%, and 75% higher mortality, respectively. In another study using the NHANES database (1988–1994), 10,750 patients were followed until 2019; the cause-specific mortality analysis showed similar proportions of death from malignancy (22.8%) and cardiovascular disease (22.1%) in MASLD (N = 3,774). Malignancy was a more common cause of death than cardiovascular disease in 167 patients with Met-ALD (28.6% vs 22%) and 361 patients with ALD (28.9% vs 17.8%) [6]. These findings highlight the carcinogenic potential of alcohol and its significance in patients with associated metabolic risk factors (22).

### Waitlist and post-transplant outcomes

In a retrospective analysis of the United Network for Organ Sharing registry, the waitlist registrations for patients with Met-ALD increased 2.9-fold and LT increased 3.3-fold from 2002 to 2022, reflective of the growing burden of this condition (23). Notably, Met-ALD exhibited worse pre-LT and post-LT outcomes compared with ALD. Waitlist removal secondary to death or clinical deterioration was more common in Met-ALD compared with patients with ALD (adjusted subhazard ratio [SHR] 1.10; *P* value: 0.007). Furthermore, post-LT outcomes in patients with Met-ALD were also poorer in Met-ALD vs ALD, with a higher risk of graft failure (SHR, 1.12; *P* value: 0.01) and patient mortality from any cause (SHR, 1.13; *P* value: 0.01) (23). In this analysis, MASLD vs ALD also had a higher risk of waitlist mortality (SHR, 1.21, *P* value < 0.001), graft failure (SHR, 1.21, *P* value < 0.001), and all-cause mortality after LT (SHR, 1.22, *P* value < 0.001) (23).

## TREATMENT OF Met-ALD

Management of Met-ALD should include control of both the risk factors, metabolic comorbidities, and alcohol use.

### Lifestyle intervention

1. **Alcohol abstinence**

As control of multiple risk factors may be difficult as the patient level, control of alcohol use is more important of the 2 risk factors and should be addressed first because this can to some extent also address metabolic issues with avoidance of empty calories from alcohol. Alcohol cessation frequently requires combined psychosocial and medical interventions. As hepatologists perceive lack of adequate training to address alcohol use disorders (AUD) in patients with liver disease (24), a multidisciplinary approach with addiction medicine experts should be taken for a holistic comprehensive patient care (7,25–27). In the future, we envision hepatology providers receiving training in addiction medicine during the gastrointestinal and/or hepatology fellowship curricula to hopefully independently provide the necessary psychosocial support and management of alcohol use in patients with liver disease including Met-ALD (28).

The approved medications by the US Food and Drug Administration (Table 1) for the treatment of AUD are disulfiram, naltrexone, and acamprosate (7). Disulfiram is an aldehyde dehydrogenase inhibitor, whose use results in unpleasant adverse effects such as flushing, nausea, and palpitations if alcohol is consumed, thus promoting alcohol cessation. It is used in highly motivated patients to abstain from alcohol who can adhere to the medication regimen. Given hepatic metabolism, there is a potential for hepatotoxicity, and the drug should be avoided in ALD patients with any spectrum of the disease (29). Naltrexone is a Mu-opioid receptor antagonist that aids with alcohol cessation by reducing the rewarding effects of alcohol. Acamprosate, on the other hand, is an NMDA receptor agonist that reduces craving and helps maintain sobriety in patients with AUD. Other non-FDA-approved medications for AUD include gabapentin, topiramate, and baclofen (30).

**Table 1.** Medications to treat alcohol use disorder

Medication	Dose	Use in ALD	Adverse effects
Naltrexone (30)	25–50 mg/d oral 380 mg/mo. IM	Observational data reported safety (31,32) and high-quality RCT needed	Diarrhea, nausea, somnolence
Acamprosate (30)	666 mg 3 times a day, oral	Can be used	Diarrhea Dose adjustment for CrCl 30–50 and avoid below 30 mL/min
Disulfiram (29)	250–500 mg loading, 250 mg/d regular oral	Can be used	Drowsiness, neuropathy, psychosis, hepatotoxicity
Topiramate (33)	Initially 25 mg/d for 4 wk, titrated every wk. Up to 300 mg/d, oral	Can be used	Paresthesia, altered taste, anorexia, difficulty concentration avoid in HE and reduce dose if Cr Cl < 70 mL/min
Baclofen (34)	Initially 5 mg 3 times/d titrate q 3–5 d to 15 mg 3 times/d, oral	Can be used	Fatigue, sleepiness, and dry mouth
Gabapentin (35)	300 mg/d initially, titrate by 300 mg q 2d to maximum 600 mg 3 times/d, oral	Possible (in case reports)	Fatigue, headache, insomnia

ALD, alcohol-associated liver disease; CrCl, creatinine clearance; RCT, randomized controlled trial.

Modified from Holbeck et al. *J Clin Translational Hepatol* (27).

Several psychosocial therapeutic modalities have been studied in AUD. Cognitive behavioral therapies and couples' therapy, among other modalities can be effective options for AUD.

**2. Weight loss**

Weight loss plays a pivotal role in controlling metabolic dysfunction (36,37). The American Association for the Study of Liver Diseases recommends at least 5% weight loss of total body weight to achieve clinically significant outcomes related to MASLD (37). Among patients with MASH, weight loss ≥5% of total body weight resolves hepatic steatosis, ≥7% of total body weight leads to MASH resolution, and ≥10% improves fibrosis stage (37). Weight loss is usually achieved through a comprehensive and stepwise approach that encompasses dietary restrictions and nutritional interventions, exercise, FDA-approved pharmacological interventions (Table 2), and bariatric surgery (38–40). These interventions have yet to be studied in patients with Met-ALD to prove their effectiveness. As bariatric surgery, especially Roux-en-Y gastric bypass procedure, has been associated with new onset or worsening of existing AUD (41,42), a close monitoring is critical in patients with Met-ALD after undergoing bariatric surgery.

**Table 2.** Medications for weight loss

Medication	Route of use	Mechanism	Metabolic improvement^a^	Adverse effects	Caution
Orlistat	Oral	Blocks gastrointestinal fat absorption	9%–10%	Oily stools and steatorrhea, flatulence	Liver disease Monitor for cholelithiasis
Phentermine	Oral	Sympathomimetic to increase norepinephrine in hypothalamus	6%–7%	Dry mouth, headache, and insomnia	Anxiety/depression
Naltrexone-bupropion	Oral	Reduce appetite and food cravings through central proopiomelanocortin neurons	6%	Nausea, vomiting, constipation, dizziness, and headache	Reduce dose in liver disease
GLP-1 agonists^b^	Subcutaneous Oral	Stimulate enteropancreatic hormones such as GLP-1, GIP, and glucagon and reduce appetite through gut-brain axis	6%–20%	Nausea, diarrhea, vomiting, abdominal pain, constipation, headache, pancreatitis	Cirrhosis Monitor for cholelithiasis

GIP, gastric inhibitory polypeptide; GLP-1, glucagon like receptor-1.

a Effects on weight, blood pressure, and lipid profile.

b Liraglutide, semaglutide (also available as oral preparation), tirzepatide.

### Optimizing cardiometabolic risk factors

Controlling other CMRFs is an essential step in the management of Met-ALD. Metabolic syndrome and its complications play a major part in the morbidity and mortality in patients with Met-ALD. With a focus of providing comprehensive care to reduce all-cause mortality in patients with CMRF, physicians should optimize control of diabetes mellitus, hypertension, and dyslipidemia (37).

**3. Medications**

In addition to therapies used for AUD, weight loss, and optimizing CMRF, newer therapies that target both aspects of Met-ALD have promising results in the improvement of liver fibrosis and reduction of steatosis and may further promote alcohol abstinence. This section will deal with critical pharmacological interventions that have potential to be useful in the treatment of Met-ALD, which are essentially drugs targeting metabolic pathways and AUD.

#### Resmetirom.

Among the most promising therapies in Met-ALD is resmetirom, the selective thyroid hormone receptor-β agonist or THR-β (43). In a phase-3 MAESTRO-NASH clinical trial, resmetirom improved liver fibrosis by at least 1 stage without worsening of MASH and resolved MASH without worsening of fibrosis in patients with MASLD (44). Overall, 88% of patients dosed with 100 mg of resmetirom achieved a ≥30% relative reduction in liver fat content as assessed by MRI-proton density fat fraction, whereas 81% receiving 80 mg did similarly (44). By contrast, only 14% in the placebo group showed similar improvements. Furthermore, a subgroup analysis of 75 patients meeting criteria for Met-ALD based on alcohol use and alcohol biomarkers (Carbohydrate-deficient transferrin and phosphatidylethanol) provided evidence for significant improvement in fibrosis and MASH. These findings suggest that resmetirom could be equally effective in patients with Met-ALD.

#### GLP-1 and gastric inhibitory polypeptide agonists.

Semaglutide and tirzepatide are GLP-1 and GLP-1/gastric inhibitory polypeptide agonists, respectively, approved for the treatment of type 2 diabetes and obesity (39). Interestingly, observational and retrospective data on the use of these medications have shown effects to reduce alcohol consumption (45). For example, in 1 retrospective analysis on 153 obese patients, 106 receiving GLP-1 (50 semaglutide and 56 tirzepatide) reported reduction in alcohol intake after starting these medications as compared with 47 patients not receiving GLP-1 (46). Patients received either semaglutide or tirzepatide, after which AUD Identification Test (AUDIT) scores were found to be lower, indicating fewer drinks per drinking episode compared with the control group (46). However, their efficacy on decreasing alcohol use in randomized controlled trials has been conflicting (47). Clearly, high-quality randomized clinical trials (www.clinicaltrials.gov) are needed to examine the benefit of GLP-1 analogs in reducing alcohol use in patients with liver disease and Met-ALD. Other than improvement in comorbidities (diabetes mellitus and obesity), data are also emerging on the liver-targeted benefits of these drugs in patients with MASH. In a phase-2 randomized clinical trial on 190 patients with MASH, semaglutide treatment for 52 weeks was superior vs placebo in resolution of MASH without fibrosis worsening (48). A preliminary data from phase 3 ESSENCE trial showed that 72 weeks of treatment with semaglutide compared with placebo improved MASH (62.9% vs 34.1%) and fibrosis (37% vs 22.5%), *P* < 0.0001 for both (49). This dual action of weight reduction and alcohol consumption reduction is of great importance in Met-ALD, addressing the key contributors to liver disease progression.

#### FGF21 analogs.

FGF21 is one of the most important metabolic regulator expressed in the liver because of alcohol consumption and has been shown to reduce alcohol consumption through its action in the brain reward centers. More precisely, FGF21 acts through receptors in the amygdala and locus coeruleus to dampen the brain response to alcohol, thereby reducing craving and consumption (50). Data from animal studies indicate that the increase in the levels of FGF21 depressed alcohol consumption and might, therefore, represent a new therapeutic target in patients with Met-ALD (50). In humans, alcohol increases levels of FGF21, and pharmacological activation may reduce the progression of alcoholic liver disease and the risk of its complications such as cirrhosis. In a systematic review addressing the efficacy of FGF21 analogs in MASLD treatment, Jeong et al identified 8 studies with 963 patients treated with FGF21, namely efruxifermin, pegbelfermin, and pegozafermin (51). FGF-21 analogs were significantly better in achieving liver histological improvements (risk ratio: 1.83, *P* = 0.001) and beneficial biochemical outcomes (improved alanine aminotransferase, aspartate aminotransferase, and triglycerides) compared with placebo, with a tolerable safety pattern. In this study, the most prevalent adverse events were injection site erythema and gastrointestinal conditions, which did not lead to serious complications or high rates of treatment discontinuation (51).

## CLINICAL TRIAL END POINTS IN Met-ALD

Addressing alcohol use in patients with Met-ALD presents several challenges in clinical trial design. Recently, an expert panel commissioned by the National Institute on Alcohol Abuse and Alcoholism (NIAAA) proposed a series of recommendations for designing clinical trials to evaluate alcohol use in liver disease. The consensus statement emphasized the importance of quantifying alcohol consumption, stratifying patients by alcohol intake and metabolic risk factors, and incorporating both pharmacological and behavioral interventions (52). Clinical trials should measure liver-related outcomes such as fibrosis progression, liver function tests, decompensation, Child-Pugh and Model of End-Stage Liver Disease scores, hospitalization, and LT-free survival. On the other hand, alcohol use outcomes include abstinence, percent of heavy drinking days, Alcohol Use Disorders Identification Test score, AUD symptom assessment, and Peth levels. End points in clinical trials on Met-ALD should include AUD outcomes and metabolic outcomes, both of which drive liver-related outcomes. Consequently, the combination of these outcomes is reflected on patient-reported outcomes and quality of life.

**Figure 2.**
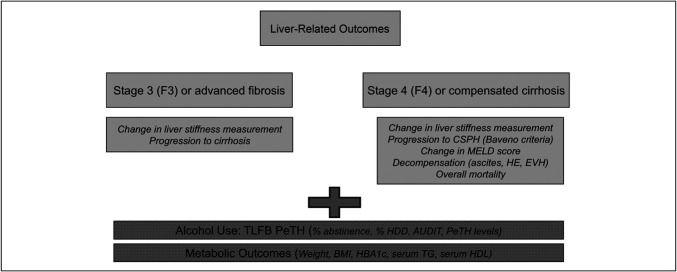
End points and outcomes for phase 2 and 3, clinical trials in patients with metabolic dysfunction-associated and alcohol-associated liver disease. AUDIT, alcohol use disorder identification test; BMI, body mass index; BSL, blood sugar level; CSPH, clinically significant portal hypertension; EVH, esophageal variceal hemorrhage; FBS, fasting blood sugar; HDD, heavy drinking days; HDL, high-density lipoprotein; HE, hepatic encephalopathy; MELD, model for end-stage liver disease; PeTH, phosphatidyl ethanol; PP, post prandial; T2-DM, type 2 diabetes mellitus; TG, triglyceride; TLFB, time line follow back.

We suggest that the patient population for Met-ALD trials should focus on patients with F3-F4 fibrosis given the fact that they have the highest rates for developing liver outcomes such as progression to cirrhosis in the F3 population and the development of decompensation in the F4 population. These patients can be identified using noninvasive tests such as vibration controled transient elastography and enhanced liver fibrosis although the performance of these noninvasive tests in the Met-ALD population requires further validation. We believe that a trial duration of 36–48 months in a large enough population should provide the power needed to detect a beneficial effect of a pharmacologic intervention. A similar duration is implemented in the MAESTRO-NASH-OUTCOMES trial (NCT05500222) although the annual event rate is expected to be lower than that in a Met-ALD population. To calculate the sample size, an understanding is needed on annual event rate, treatment duration, and effect size of the intervention. We highly encourage the pharmaceutical sponsors to consider having a biomarker strategy in their Met-ALD trials that can predict baseline severity, assess risk for outcomes, and determine response to the intervention. Adaptive trial design to transition from phase 2 to phase 3 could be considered. A summary of these recommendations is provided in Figure 2.

## CONCLUSION

Metabolic and ALD (Met-ALD) is a unique entity within the spectrum of SLD because of the interplay between metabolic dysfunction and moderate alcohol consumption. Being defined as a separate entity from MASLD and ALD requires better understanding of Met-ALD diagnosis, staging, prognostication, and treatment. Notably, it also carries different risks of liver fibrosis, cirrhosis, and HCC. Treatment of Met-ALD is multifaceted and relies on addressing both alcohol use and metabolic risk factors. Consequently, alcohol abstinence and weight loss are key in Met-ALD management. Moreover, novel therapeutics, such as resmetirom, hold potential for both metabolic and alcohol-related liver injury, whereas newer agents, including semaglutide, tirzepatide, and FGF21 analogs, represent new opportunities in the treatment of alcohol use, metabolic dysfunction, and liver outcomes. Suggested clinical trials' end points include ALD, AUD, CMRF, patient-reported outcomes, and quality of life. In addition, research should try to provide data on PeTH levels and other biomarkers to define different SLD populations. Furthermore, clinical management and research will similarly rely on Met-ALD-specialized therapies as the key to better patient outcomes in this increasingly prevalent population.

## CONFLICTS OF INTEREST

**Guarantor of the article:** Ashwani K. Singal, MD, MS, FACG, FAASLD, AGAF.

**Specific author contributions:** N.D. and A.I. performed literature search and wrote the manuscript draft. A.K.S. conceived the idea for the article. A.K.S. and N.A. supervised and contributed to intellectual input. All the authors reviewed the final version and approved for submission.

**Financial support:** Kentucky Medicaid (SUP26-C5531), NIH (P20 GM103436 supplement), and NIAAA (U01 AA026980-06) to AKS.

**Potential competing interests:** N.A. has received grant/research support from 89bio, Akero Therapeutics, Arbutus Biopharma, AstraZeneca, BioAge, Boehringer Ingelheim, Bristol Myers Squibb, Corcept Therapeutics, CymaBay Therapeutics, *DSM*, Galectin Therapeutics, Genentech, Genfit, Gilead Sciences, Healio, Hepagene Therapeutics, Intercept Pharmaceuticals, Inventiva Pharma, Ionis Pharmaceuticals, Ipsen, Lilly, Madrigal Pharmaceuticals, Merck, NGM Biopharmaceuticals, Noom, NorthSea Therapeutics, Novo Nordisk, Perspectum, Pfizer, PharmaIN, Poxel, Viking Therapeutics, and Zydus Pharmaceuticals; reports speaker's fees from AbbVie, AstraZeneca, Echosens, Gilead Sciences, Intercept Pharmaceuticals, Ipsen, Madrigal Pharmaceuticals, and Perspectum; and reports consulting for 89bio, Akero, Boehringer Ingelheim, Echosens, Fibronostics, Gilead Sciences, Intercept Pharmaceuticals, Ipsen, Madrigal Pharmaceuticals, NorthSea Therapeutics, Novo Nordisk, Perspectum, Pfizer, and Regeneron. AKS reports (a) Speaker's Bureau and Writing for Medscape Gastroenterology, Chronic Liver Disease Foundation, Expert Perspectives, Gastro Endo News, Dynamed, Medical Education Speakers Network, Up-to-Date, Madrigal Pharmaceuticals; (b) Grants to institution from ACG, NIH (NIAAA, NIDDK, and NIGMS), and DHHS; and (c) Consultant on the SBIR grant for Pleiogenix pharmaceuticals.
